# Intraspecies Polymorphisms in the Lipophosphoglycan of *L. braziliensis* Differentially Modulate Macrophage Activation via TLR4

**DOI:** 10.3389/fcimb.2019.00240

**Published:** 2019-07-10

**Authors:** Tamara da Silva Vieira, Jeronimo Nunes Rugani, Paula Monalisa Nogueira, Ana Cláudia Torrecilhas, Celia Maria Ferreira Gontijo, Albert Descoteaux, Rodrigo Pedro Soares

**Affiliations:** ^1^Instituto René Rachou, Fundação Oswaldo Cruz - FIOCRUZ, Belo Horizonte, Brazil; ^2^Universidade Federal de São Paulo, UNIFESP, Diadema, Brazil; ^3^INRS-Institut Armand-Frappier, Université du Québec, Laval, QC, Canada

**Keywords:** *Leishmania braziliensis*, virulence, lipophosphoglycan, clinical forms, macrophages, innate immunity

## Abstract

Lipophosphoglycan (LPG) is the major *Leishmania* surface glycoconjugate having importance during the host-parasite interface. *Leishmania* (*Viannia*) *braziliensis* displays a spectrum of clinical forms including: typical cutaneous leishmaniasis (TL), mucocutaneous (ML), and atypical lesions (AL). Those variations in the immunopathology may be a result of intraspecies polymorphisms in the parasite's virulence factors. In this context, we evaluated the role of LPG of strains originated from patients with different clinical manifestations and the sandfly vector. Six isolates of *L. braziliensis* were used: M2903, RR051 and RR418 (TL), RR410 (AL), M15991 (ML), and M8401 (vector). LPGs were extracted and purified by hydrophobic interaction. Peritoneal macrophages from C57BL/6 and respective knock-outs (TLR2^−/−^ and TLR-4^−/−^) were primed with IFN-γ and exposed to different LPGs for nitric oxide (NO) and cytokine production (IL-1β, IL-6, IL-12, and TNF-α). LPGs differentially activated the production of NO and cytokines via TLR4. In order to ascertain if such functional variations were related to intraspecies polymorphisms in the LPG, the purified glycoconjugates were subjected to western blot with specific LPG antibodies (CA7AE and LT22). Based on antibody reactivity preliminary variations in the repeat units were detected. To confirm these findings, LPGs were depolymerized for purification of repeat units. After thin layer chromatography, intraspecies polymorphisms were confirmed especially in the type and/size of sugars branching-off the repeat units motif. In conclusion, different isolates of *L. braziliensis* from different clinical forms and hosts possess polymorphisms in their LPGs that functionally affected macrophage responses.

## Introduction

The Lipophosphoglycan (LPG) is the most studied glycoconjugate expressed by *Leishmania*. It is a pathogen-associated molecular pattern (PAMP) covering the entire promastigote surface and flagellum and is implicated in a wide variety of events during the interaction of the parasite with vertebrate and invertebrate hosts. Inter and intraspecies polymorphisms in the LPGs and glycoinositolphospholipids (GIPLs) structures from several species have been reported as important for the virulence mechanisms especially during the innate immune compartment (Becker et al., 2003; de Veer et al., 2003; Spath et al., 2003; De Assis et al., 2012).

LPG comprises four distinct domains: a lipid anchor consisting of 1-*O*-alkyl-2-*lyso*-phosphatidylinositol, a central core represented by a heptassacaride Gal (α1,6)Gal(α1 (3)Gal_f_(β1,3)[Glc(α1)-PO_4_]Man(α1,3)Man(α1,4)-GlcN(α1), a conserved backbone of repeating Gal(β1,4)Man(α1)-PO_4_ units and a terminal oligosaccharide named cap (Turco and Descoteaux, 1992). Most of the polymorphisms in the structure of LPG are located in the repeat units and cap structures. Several functions have been attributed to LPG including: inhibition of phagosome maturation and acidification (Desjardins and Descoteaux, 1997; Vinet et al., 2009), inhibition of PKC (Holm et al., 2001), induction of PKR (de Carvalho Vivarini et al., 2011), induction of extracellular neutrophil networks (NETs) (Guimaraes-Costa et al., 2009; Gabriel et al., 2010), induction of heme-oxygenase I (Luz et al., 2012), LTB4 (Tavares et al., 2014), PPAR-γ (Lima et al., 2017) and modulation of NO/cytokines, MAPKs and NF-kB translocation, TLR2/TLR4 agonist (De Assis et al., 2012; Ibraim et al., 2013; Paranaíba et al., 2015; Nogueira et al., 2016). However, there are still uncertainties in how intraspecies structural and compositional polymorphisms of LPG affect parasite virulence.

Polymorphisms in the repeat units of LPGs have already been reported in several species/strains of *Leishmania* from Old and New World (De Assis et al., 2012). Studies on intraspecies LPG polymorphisms are scarce and used a limited number of strains. For example, in Old World strains of *L. donovani* (1S-1D and MONGI) (Mahoney et al., 1999), *L. major* (FV1 and LV39) (Dobson et al., 2003) and *L. tropica* (L747, L810, and L863) (Soares et al., 2004). On the other hand, most of the studies have characterized LPG polymorphisms in New World strains including: *L. infantum* (14 strains) (Coelho-Finamore et al., 2011), *L. enriettii* (L88 and Cobaia) (Paranaíba et al., 2015), and *L. amazonensis* (PH8 and Josefa) (Nogueira et al., 2017). Studies on the glycobiology of *L. braziliensis* started in 2005, where the structure of the LPG strain M2903 was characterized as important during the interaction with the sandfly vector (Soares et al., 2005, 2010). In the procyclic form it has no side-chains branching-off the repeat units, whereas in the metacyclic stage it possesses 1-2 β-glucose side-chains (Soares et al., 2005). In mouse macrophages, *L. braziliensis* LPG was more pro-inflammatory than that of *L. infantum*. It was a stronger TLR2/TLR4 agonist inducing NO and cytokine production and NF-κB translocation (Ibraim et al., 2013). It is already known that intraspecies polymorphisms in the *L. infantum* LPG results in differential production of NO by murine macrophages (Coelho-Finamore et al., 2011). In this species, there are three types of LPG (I, II, and III) depending on the presence/absence of β-glucose side chains. Although *L. braziliensis* LPG is a very pro-inflammatory PAMP among different *Leishmania* species, nothing is known about intraspecies polymorphisms in this glycoconjugate.

In Americas, *L. braziliensis* causes either single cutaneous lesions (TL) at the site of the bite or metastasizes to the oronasopharyngeal mucosa (ML). Some lesions characterized as atypical (AL) of *L. braziliensis* have been previously reported by Guimarães et al. (2009) and more recently by Quaresma et al. (2018). Interestingly, some lesions are somewhat unusual, hindering correct clinical diagnosis. Those are so called atypical lesions (AL): they are lupoid, verrucous sometimes resembling to tumors that do not fit in the regular shape of the TL lesions. Previous findings showed a differential expression of cytokines/chemokines in AL patients compared to TL patients (Costa-Silva et al., 2014). AL lesions are more difficult to heal, and this was probably due to natural resistance to Sb-based chemotherapeutic schemes (Rugani et al., 2018). However, it is still unknown if LPGs from *L. braziliensis* may be responsible for the virulence degrees observed in several strains of this species.

Here, we intend to investigate if the intraspecies variability in LPGs from different clinical forms and hosts are associated to the immunopathological events in *L. braziliensis*.

## Materials and Methods

### Ethics Statement

All animals were handled in strict accordance with animal practice as defined by Internal Ethics Committee in Animal Experimentation (CEUA) of Fundação Oswaldo Cruz (FIOCRUZ), Belo Horizonte, Minas Gerais (MG), Brazil (Protocol L-32/16). The procedures for strains isolation from humans were carried out in accordance with the recommendations of the National Committee for Research Ethics (CONEP # 355/2008).

### Cell Culture

*Leishmania braziliensis* reference strains were used including: MHOM/BR/75/M2903 (TL), MHOM/BR/1996/M15991 (ML), IWELL/BR/1981/M8401 (vector). Other isolates included (RR051 and RR418) (TL) and RR410 (AL). Those strains were isolated from human patients in the Xakriabá indigenous community located in São João das Missões, Minas Gerais State, Brazil (Quaresma et al., 2018). Those strains were previously typed as reported (Rugani et al., 2018). Starter cultures of promastigotes were grown in supplemented Medium 199 as reported elsewhere (Soares et al., 2002).

### Extraction and Purification of LPG

LPG extraction was performed as described elsewhere with solvent E (H_2_O/ethanol/diethylether/pyridine/NH_4_OH; 15:15:5:1:0.017) after a sequential organic solvent extraction. For purification, the solvent E extract was dried under N_2_ evaporation, resuspended in 2 mL of 0.1N acetic acid/0.1 M NaCl, and applied onto a column with 2 mL of phenyl-Sepharose, equilibrated in the same buffer (Soares et al., 2002, 2004).

### Macrophages, Nitrite, and Cytokines

Thioglycollate-elicited peritoneal macrophages were extracted from C57BL/6 and C57BL/6 (TLR2 and TLR4 knockouts) as previously reported (Ibraim et al., 2013; Nogueira et al., 2016). Briefly, recovered cells (3 × 10^5^ cells/well) were washed with fresh RPMI and cultured in the same medium supplemented with 2 mM glutamine, 50 U/ml of penicillin and 50 μg/mL streptomycin, 10% Fetal Bovine Serum in 96-well culture plates (37°C, 5% CO_2_). Cells were primed with gamma interferon (IFN-γ) (3 IU/mL) for 18 h prior to incubation with LPGs (10 μg/mL) from all strains and controls for 48 h. Those included LPS (100 ng/mL, positive) and medium (negative). The nitrite concentration was measured by Griess reaction. For cytokine detection, supernatants were collected and IL-1β, IL-6, IL-12, and TNF-α were determined using BD CBA Mouse Cytokine assay kits according to the manufacturer's specifications (BD Biosciences, CA, USA). Flow cytometry measurements were performed on a FACS Calibur flow cytometry (BD Bioscience, Mountain View, CA, USA). Cell-Quest TM software package provided by the manufacturer was used for data acquisition and the FlowJo software 7.6.4 (Tree Star Inc., Ashland, OR, USA) was used for data analysis (Nogueira et al., 2016).

### Western Blot

Purified LPGs (10 μg) were resolved by SDS-PAGE electrophoresis and transferred to nitrocellulose membrane. Blots were probed with monoclonal antibody (mAb) CA7AE (1:1,000), that recognizes the unsubstituted Gal(β1,4)Man(α1)-PO_4_ repeat units (Tolson et al., 1989) and LT22 (1:1,000) that recognizes β-glucose/β-galactose side chains. After washing in PBS (3 × 5 min), the membrane was incubated for 1 h with antimouse IgG conjugated with peroxidase (1:10,000) and the reaction was visualized using luminol (Soares et al., 2005; Nogueira et al., 2017).

### Biochemical Analysis

Promastigotes were radiolabeled during stationary phase 1.0 × 10^8^-10^9^ cells/mL with 90 uCi/ml_of [6-^3^H]Gal at 26°C for 8 h as previously reported (Soares et al., 2005). [3H-Gal]-LPG was extracted and purified as described above. LPGs were depolymerized by mild acid hydrolysis (0.02 N HCl, 100°C, 5 min) in order to separate the repeating units and caps. Samples were subjected to the butanol: water partition (1:2) to remove core-PI motifs. Purified repeat units were recovered and subjected to enzymatic treatments with alkaline phosphatase prior to glycosidases (Mahoney et al., 1999). After enzymatic treatments, samples were desalted through a two-layered column of AG50W-X12(H+) over AG1-X8 (acetate). Phosphorylated oligosaccharides were treated with alkaline phosphatase in 15 mM Tris buffer, pH 9.0 (1 U, 16 h, 37°C). Neutral oligosaccharides were treated with sweet almond β-glucosidase in 200 mM ammonium acetate buffer, pH 5.0 (1 U, 16 h, 37°C). The repeat units treated with β-glucosidase were subject to thin layer chromatography technique (TLC). Samples were applied on silica plates and run in butanol pyridine water (6:4:3) solution for 20 h. Quantification of the radioactivity were performed using the Tri-Carb-1600 TR (Soares et al., 2002).

### Data Analyses

Statistical analyses and graphics construction were performed using one-way ANOVA test with Software GraphPad Prism 6.0 (GraphPadSoftware Inc., San Diego, CA, EUA). The analyses were done after normality test of Kolmogorov-Smirnov. *P* < 0.05 was considered significant.

## Results

### Macrophage Activation

**Figure 1:**
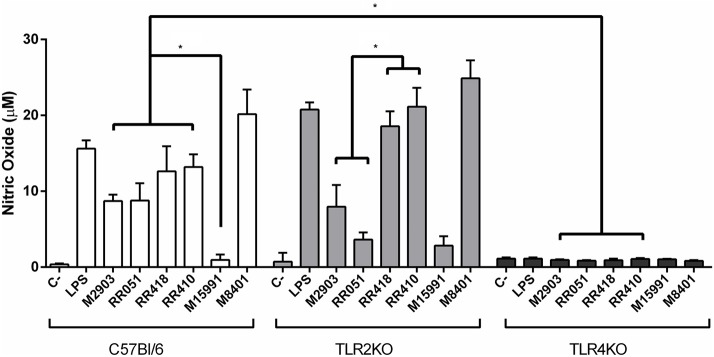
Nitrite production by IFN-γ primed macrophages stimulated with *L. braziliensis* LPGs from patients with different clinical manifestations and the sandfly vector. Cells were pre-incubated with IFN-γ (3 IU/mL) for the 18 h prior to LPG exposure (10 μg/mL) and LPS (100 ng/mL, positive control) for 48 h. Fresh medium alone was used as negative control. Nitrite concentration was measured by Griess reaction. C, negative control; M2903, RR051, and RR418, *L. braziliensis* LPG isolated from typical lesions; RR410, LPG of *L. braziliensis* from atypical lesion; M15991, LPG isolated from mucocutaneous lesion; M8401, *L. braziliensis* LPG isolated from sandfly. *P < 0.05 was considered significant.

**Figure 2:**
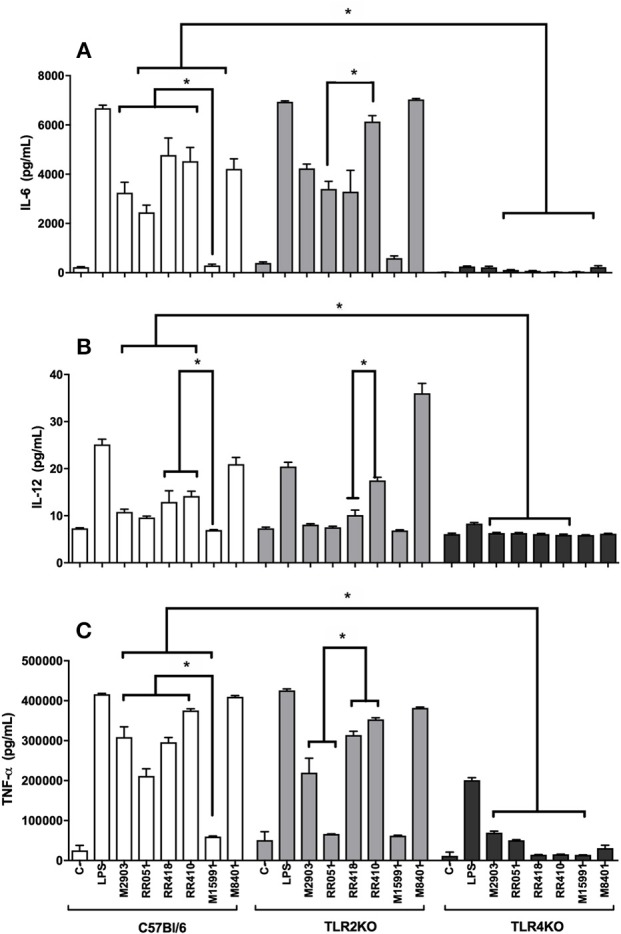
Cytokine production by IFN-γ primed macrophages stimulated with *L. brazilienis* LPGs from patients with different clinical manifestations and the sandfly vector. Cells were pre-incubated with IFN-γ (3 IU/mL) for the 18 h prior to LPG exposure (10 μg/mL) and LPS (100 ng/mL, positive control) for 48 h. Fresh medium alone was used as negative control. Cytokine concentrations of IL-6 **(A)**, IL-12 **(B)**, and TNF-α **(C)** were determined by flow cytometry. C, negative control; M2903, RR051, and RR418, *L. braziliensis* LPG isolated from typical lesions; RR410, LPG of *L. braziliensis* from atypical lesion; M15991, LPG isolated from mucocutaneous lesion; M8401, *L. braziliensis* LPG isolated from sandfly. *P < 0.05 was considered significant.

Murine macrophages were exposed to LPGs from different strains to evaluate the impact on the innate immune response. In general, the various LPGs induced NO, IL-6, IL-12, and TNF-α production preferentially via TLR4 (Figures 1, 2). However, this production varied among *L. braziliensis* strains. For example, LPG from M15991strain (ML) did not induce considerable levels of NO and cytokines. On the other hand, the LPG from the vector strain (M8401) was very proinflammatory inducing NO and cytokine levels similar to LPS (Figures 1, 2A–C). In general, LPGs from AL/TL strains RR410/RR418 induced higher levels of NO and cytokines than LPG from TL strains M2903, RR051, and M15991 (ML) in the TLR2 KO but not WT macrophages (Figures 1, 2A–C). No detectable IL-1β production was induced by the different LPGs used in this study (data not shown).

### Biochemical Analysis

**Figure 3:**
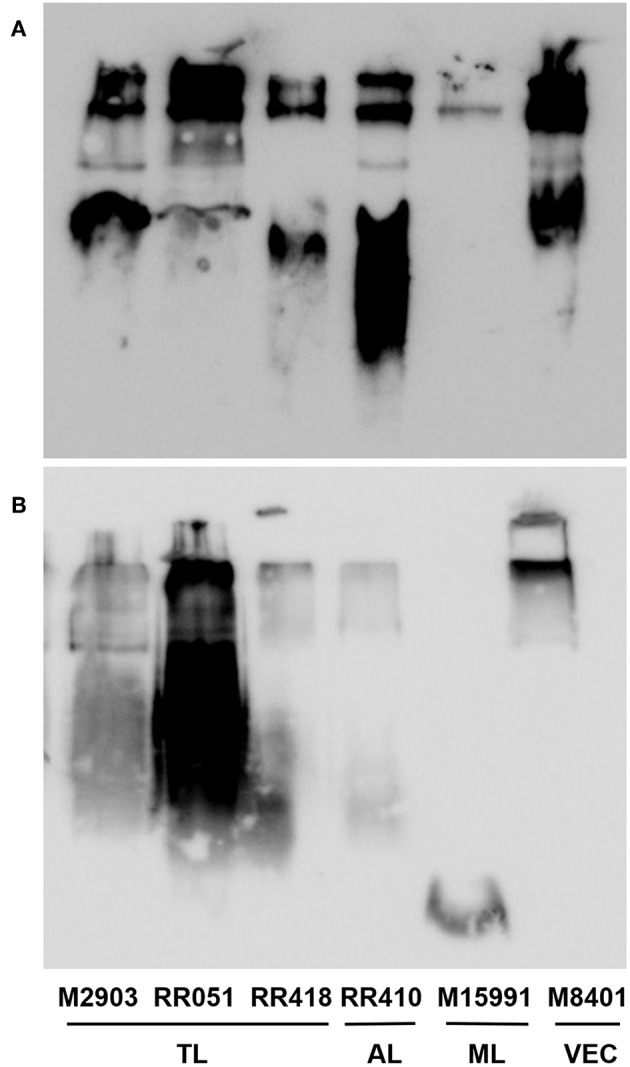
Western blot of purified lipophosphoglycan (LPG). Purified LPG (10 μg per lane) from promastigotes of *L. braziliensis* strains (M2903, RR051, RR418, RR410, M15991, and M8401) were incubated with the antibody CA7AE (1:1,000) **(A)** and LT22 (1:1,000) **(B)**. LPG purified from *L. braziliensis* M2903 strain was used as positive control. TL, Typical lesions; AL, Atypical lesions; ML, Mucocutaneous strain; VEC, Vector strain.

**Figure 4:**
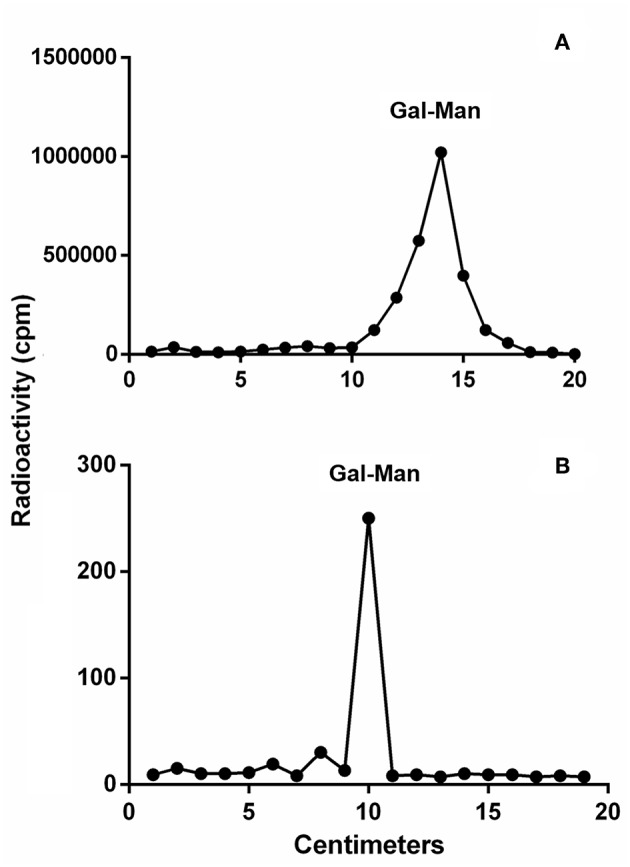
Thin layer chromatography (TLC) of dephosphorylated [^3^H]Gal-repeat units obtained from different *L. braziliensis* LPGs. **(A)** M15991 repeat units and **(B)** M8401 repeat units. Gal, galactose; Man, mannose.

**Figure 5:**
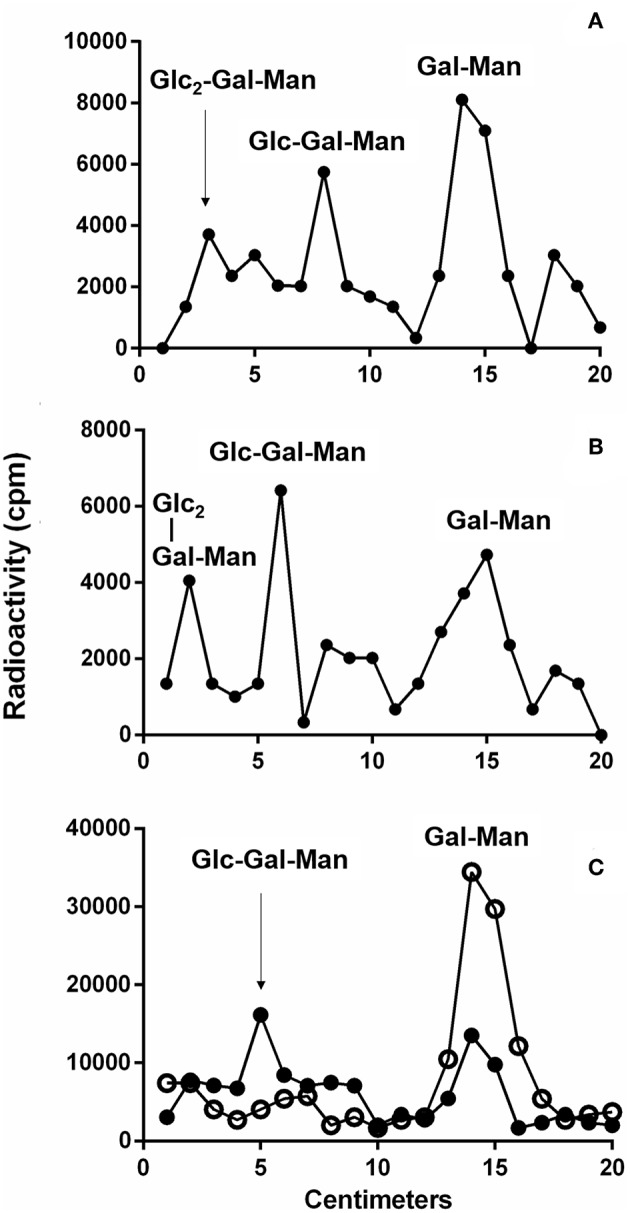
Thin layer chromatography (TLC) of dephosphorylated [^3^H]Gal-repeat units obtained from different *L. braziliensis* LPGs. **(A)** M2903 repeat units, **(B)** RR410, and **(C)** RR051 treated (open circles) or not (closed circles) with β-glucosidase. Glc, glucose; Gal, galactose; Man, mannose.

To detect if variations in macrophage responses could be functionally attributed to intraspecies polymorphisms among *L. braziliensis* LPGs, we performed a biochemical analysis of these molecules. First, a preliminary analysis using western blot with specific antibodies was conducted. In general, both antibodies recognized all purified LPGs confirming the success of the purification process. However, based on the profiles, the smears suggest the existence of polymorphisms (Figures 3A,B). For CA7AE, all LPGs were recognized by this antibody confirming the existence of Gal(β1,4)Man(α1)-PO_4_ motifs common to all LPGs (Soares et al., 2005). Different from CA7AE, LT22 exhibited a more evident polymorphisms among strains. For example, RR051 followed by M2903 strain were strongly recognized by this antibody. Those data suggest the existence of sugars branching-off the repeat units in some of the strains. Interestingly, M15991 strain, who was weakly recognized by CA7AE, was also detected very poorly by LT22. Although the western blot analyses suggest the existence of polymorphisms in the repeat units, a deeper biochemical analysis using TLC was required. To this end, purified LPGs were depolymerized and subjected to TLC analysis. The repeat units from the M15991 (ML) and M8401 (vector) strains were similar and devoid of side-chains exhibiting only a disaccharide peak (Figures 4A,B). As expected, the repeat units of M2903 LPG (control) exhibited a di-, tri- and tetrasaccharide as previously reported (Soares et al., 2005) (Figure 5A). This profile was also observed for AL isolate RR410 (Figure 5B). The repeat units of RR051 isolate exhibited a tri- and a disaccharide (Figure 5C, closed circles). In order to confirm if the side-chains were composed by β-glucose residues that could justify its reactivity to LT22 (Figure 3B), treatment with β-glucosidase was performed. After enzymatic treatment, the trisaccharide disappeared and the disaccharide peak increased confirming the presence of β-glucoses as side-chains (Figure 5C, open circles). A similar result was observed for strain RR418 (data not shown). Altogether, those data indicate the presence of side-chains in the LPG repeat units of those strains.

## Discussion

Several factors may be related to the different levels of virulence among strains of a given *Leishmania* species. In the case of *L. braziliensis*, this species causes several clinical manifestations ranging from single lesions to atypical and/or severe mucocutaneous forms (Guimarães et al., 2016; Quaresma et al., 2018). Here, we investigated the role of LPG during the activation of macrophages from the innate immune compartment. An early and successful activation of this compartment is important for the fate of the acquired immune responses especially in parasitic diseases caused by Protozoa (Gazzinelli et al., 2004). Early studies with Old World *Leishmania* species already reported the role of LPG for the induction of cytokines, NO and MAPKs (Brittingham and Mosser, 1996; Feng et al., 1999). This triggered a lot of interest in establishing the TLRs involved in the innate immune responses in the hosts [revised by (Tuon et al., 2008)]. In this context, *L. major* LPG was the first TLR2 agonist reported for human natural-killer (NK) cells and murine macrophages. This activation triggers the production of TNF-γ and IFN-γ via MyD88 and is dependent on the integrity of the lipid anchor (Becker et al., 2003; de Veer et al., 2003). Consistent with those results, the integrity of the lipid anchor of *L. infantum* LPG was also required for the activation of PPAR-γ (Lima et al., 2017). In *L. braziliensis* (M2903 strain), it was previously reported that its LPG activates NO and cytokines production via TLR4/TLR2. A distinguishing feature of this LPG is the very pro-inflammatory profile compared to that of *L. infantum* (Ibraim et al., 2013). Here, this pro-inflammatory effect remained, but other strains (RR418, RR410, and M8401) exhibited a higher ability to induce NO and cytokines than M2903 especially in the TLR2 knockout. However, those differences were not observed in the WT. Those data confirmed that LPGs from different strains/isolates have variable immuno-modulatory activities toward murine macrophages. Based on our data, this activation was mediated by TLR4, similar to other dermotropic species such as *L. amazonensis* and *L. enriettii* (Paranaíba et al., 2015; Nogueira et al., 2016).

To investigate whether LPG polymorphisms correlated with clinical forms of leishmaniasis, macrophages were stimulated with glycoconjugates purified from *L. braziliensis* from different patients. The LPG from AL strain (RR410) induced a high NO and cytokine production than most of TL and ML strains. Interestingly, the LPG isolated from a vector strain (M8401), exhibited the highest pro-inflammatory activity, comparable to that of LPS. Our results confirm those in the literature that murine macrophages are able to produce NO in response to LPG, and that this production is variable among species/strains (Coelho-Finamore et al., 2011; Ibraim et al., 2013; Paranaíba et al., 2015; Nogueira et al., 2016). Cytokines are very important during immunopathology of Leishmaniasis. For example, IL-1β and IL-6 are important proinflammatory cytokines acting on endothelial cells by increasing the number of adhesion molecules and migration of leukocytes to the site of inflammation. This favor tissue damage by increasing inflammation by attracting and activating neutrophils (Castellucci et al., 2006; Boaventura et al., 2010; Sallusto et al., 2012). In our study, an important induction of IL-6 was noticed in most of the strains, suggesting the role of *L. braziliensis* LPG in this process. On the other hand, the reference strain from the mucocutaneous lesion (M15991) induced a very low production of NO and cytokines, a response similar to that of *L. infantum* LPG (Ibraim et al., 2013). Many aspects of the immunopathology of ML strains are still unknown, especially those related to metastasis from the bite site to the oropharyngeal mucosa. This low proinflammatory potential of the LPG from the ML strain strongly suggests that other molecules such as GP63, GIPLs, and PPGs could be important during macrophage response (De Assis et al., 2012). Consistent with this, M15991 strain does not possesses LRV1 (*Leishmaniavirus*) (Macedo et al., 2016), reinforcing that other strain-specific factors and perhaps from the host could be primarily involved in the immunopathology of ML. Interestingly, there was increased production of NO, IL-6, and TNF-α by the isolated strain from an atypical lesion (AL). AL lesions exhibited an aspect macroscopically different from the common TL lesion (ulcerated with elevated borders) (Costa-Silva et al., 2014; Quaresma et al., 2018). In general, the LPG of the RR410 strain was more pro-inflammatory than those of the TL/ML lesions mainly for the TLR2 knockout (IL-6 and IL-12). This reinforces that other factors such as genetics could be responsible for these unusual forms. Recently, it was reported that AL causing strains have a SNP in the 1,300 bp hsp70 gene fragment that clustered them in a group separated from TL/ML strains (Quaresma et al., 2018). Altogether our data show that the mechanisms underlying macrophage modulation by LPG clearly vary among strains/isolates of *L. braziliensis* and are not easily correlated with pathology.

To investigate whether those differences could be due to polymorphisms in the LPG, preliminary analyzes using western-blot were performed. All LPGs were recognized by the CA7AE antibody. The LPG from M15991 strain was weakly recognized by this antibody. However, most LPGs were recognized by this mAb suggesting that they possess most of unsubstituted repeat units. This feature is commonly observed in *L. infantum* type I LPG (10 strains), *L. enriettii* (two strains), *Leishmania shawi*, and *L. donovani* (Sudan strain) (Sacks et al., 1995; Coelho-Finamore et al., 2011; Paranaíba et al., 2015; Passero et al., 2015). On the other hand, the presence of side-chains suggestive of glucoses (LT22 positive) was strongly detected in the LPG from RR051strain followed by M2903. This is consistent with the literature that the LPG of M2903 (positive control) from stationary phase possess 1-2 β-glucose side-chains (Soares et al., 2005). Those sugars are also observed in the LPG from *L. infantum* (strains PP75 and BH46), *L. amazonensis* (PH8 strain), and *L. donovani* (Mongi strain) (Mahoney et al., 1999; Soares et al., 2002; Coelho-Finamore et al., 2011; Nogueira et al., 2017). The other strains (RR418, RR410. M15991, and M8401) exhibited lower recognition by this mAb suggesting that some of the repeat units could be substituted with side-chains and this should be confirmed by TLC analysis. Those data suggest intraspecies polymorphisms in the purified repeat units from *L. braziliensis* LPGs.

To detect polymorphisms in the repeating units, we chose five *L. braziliensis* LPGs displaying different patterns, as assessed by western blots. LPGs were radioactively labeled to increase sensitivity, since *L. braziliensis* express lesser amounts of this glycoconjugate than other *Leishmania* species (Soares et al., 2005). The results indicated evident polymorphisms in the repeat units. The strains M15991 and M8401 possess LPGs without side chain confirming their basic structure of Gal(β1,4)Man(α1)-PO_4_ (Sacks et al., 1995). Although LPG expressed by strain M15991 was weakly recognized by CA7AE, it exhibited a clear disaccharide peak confirming the existence of unbranched repeating units. As expected, TLC analysis of LPG from strain M2903 (control) confirmed the existence of di-, tri- and tetrasaccharides as previously described (Soares et al., 2005). Confirming the western-blot data, in addition to a disaccharide, the LPG of RR051 also exhibited a tetrasaccharide peak. After enzymatic treatment with β-glucosidase this peak disappeared followed by an increase in the disaccharide peak. This confirmed the existence of β-glucoses as side-chains. Similar results were also observed for RR418 (data not shown), *L. infantum* and *L. mexicana* (Ilg et al., 1992; Soares et al., 2002). Those data confirmed that β-glucoses are the most common side chains found in the LPGs from New World species of *Leishmania*.

Those polymorphisms were functionally compared during macrophage studies. Depending on the cytokine/NO a correlation could be established. For example, the production of NO, TNF-α and IL-6 by WT murine macrophages was higher in response to glucosylated LPGs (M2903, RR051, and RR410) than that of unbranched LPG from M15991. However, for M8401 this correlation could not be established. It has an unbranched LPG but alike M15991 it exhibited a high pro-inflammatory activity. It is not likely that this feature is due to the presence of side-chains since it does not have it. Conversely, we could postulate that this could be due to a longer LPG size. This was already reported in another dermotropic species *L. enriettii*. Two strains of this species were compared and the LPG of L88 strain (longer size) was more pro-inflammatory than that of the Cobaia strain (short size) (Paranaíba et al., 2015). Those data suggested that LPG qualitative variations either in sugars branching-off the repeating units or size could differentially modulate macrophage responses. However, this is dependent on species/strains. For example, in *L. infantum* the presence of glycosylated LPGs (strains PP75 and BH46) triggered a higher NO production compared to unsubstituted (type I) LPGs (Coelho-Finamore et al., 2011). On the other hand, in *L. amazonensis*, polymorphisms in the sugar side-chains were not important for macrophage activation and this could be attributed to the lipid anchor (Nogueira et al., 2017).

In conclusion, our study showed that strains/isolates of *L. braziliensis* differentially activated murine macrophages via TLR4. We found considerable structural and compositional polymorphisms in the side-chains of these LPGs that could affect interaction with this TLR, although a clear correlation between structure and function could not be established. *Leishmania*-macrophage interaction process is very complex involving parasite and immune cells molecules. Our data reinforce the idea that not only LPG but other molecules play an important role during the interface parasite-host and perhaps affect the immunopathology. Based on our work, differences in virulence/pathogenicity are strongly dependent on species/strains. This is surely the case of the *L. braziliensis*, a species causing a spectrum of dermotropic diseases.

## Data Availability

The raw data supporting the conclusions of this manuscript will be made available by the authors, without undue reservation, to any qualified researcher.

## Author Contributions

RS, AD, CG, and AT designed the experiments. JR, TV, PN, and AT performed the experiments. PN, TV, AD, CG, and RS analyzed the data. All authors contributed for the manuscript.

### Conflict of Interest Statement

The authors declare that the research was conducted in the absence of any commercial or financial relationships that could be construed as a potential conflict of interest.

## References

[B1] Becker I. Salaiza N. Aguirre M. Delgado J. Carrillo-Carrasco N. Kobeh L. G.. (2003). Leishmania lipophosphoglycan (LPG) activates NK cells through toll-like receptor-2. Mol. Biochem. Parasitol. 130, 65–74. 10.1016/S0166-6851(03)00160-9 12946842

[B2] Boaventura V. S. Santos C. S. Cardoso C. R. De Andrade J. Dos Santos W. L. C. Clarêncio J.. (2010). Human mucosal leishmaniasis: neutrophils infiltrate areas of tissue damage that express high levels of Th17-related cytokines. Eur. J. Immunol. 40, 2830–2836. 10.1002/eji.200940115 20812234

[B3] Brittingham A. Mosser D. (1996). Exploitation of the complement system by *Leishmania promastigotes*. Parasitol. Today 12, 444–447. 10.1016/0169-4758(96)10067-3 15275279

[B4] Castellucci L. Menezes E. Oliveira J. Magalhaes A. Guimaraes L. H. Lessa M. (2006). IL6-174 G/C promoter polymorphism influences susceptibility to mucosal but not localized cutaneous leishmaniasis in Brazil. J. Infect. Dis. 194, 519–527. 10.1086/505504 16845637

[B5] Coelho-Finamore J. M. Freitas V. C. Assis R. R. Melo M. N. Novozhilova N. Secundino N. F.. (2011). *Leishmania infantum*: lipophosphoglycan intraspecific variation and interaction with vertebrate and invertebrate hosts. Int. J. Parasitol. 41, 333–342. 10.1016/j.ijpara.2010.10.004 21118695

[B6] Costa-Silva M. F. Gomes L. I. Martins-Filho O. A. Rodrigues-Silva R. Freire J. de M. FláviaQuaresma P.. (2014). Gene expression profile of cytokines and chemokines in skin lesions from Brazilian Indians with localized cutaneous leishmaniasis. Mol. Immunol. 57, 74–85. 10.1016/j.molimm.2013.08.008 24084096

[B7] De Assis R. R. Ibraim I. C. Nogueira P. M. Soares R. P. Turco S. J. (2012). Glycoconjugates in new world species of leishmania: polymorphisms in lipophosphoglycan and glycoinositolphospholipids and interaction with hosts. Biochim. Biophys. Acta Gen. Subj. 1820, 1354–1365. 10.1016/j.bbagen.2011.11.001 22093608

[B8] de Carvalho Vivarini A. Pereira R. d. M. S. Dias Teixeira K. L. Calegari-Silva T. C. Bellio M. Dalastra Laurenti M. (2011). Human cutaneous leishmaniasis: interferon-dependent expression of double-stranded RNA-dependent protein kinase (PKR) via TLR2. FASEB J. 25, 4162–4173. 10.1096/fj.11-185165 21846836

[B9] de Veer M. J. Curtis J. M. Baldwin T. M. DiDonato J. A. Sexton A. McConville M. J.. (2003). MyD88 is essential for clearance of *Leishmania major*: possible role for lipophosphoglycan and Toll-like receptor 2 signaling. Eur. J. Immunol. 33, 2822–2831. 10.1002/eji.200324128 14515266

[B10] Desjardins M. Descoteaux A. (1997). Inhibition of phagolysosomal biogenesis by the leishmania lipophosphoglycan. J. Exp. Med. 185, 2061–2068. 9182677 10.1084/jem.185.12.2061 PMC2196352

[B11] Dobson D. E. Mengeling B. J. Cilmi S. Hickerson S. Turco S. J. Beverley S. M. (2003). Identification of genes encoding arabinosyltransferases (SCA) mediating developmental modifications of lipophosphoglycan required for sand fly transmission of *Leishmania major*. J. Biol. Chem. 278, 28840–28848. 10.1074/jbc.M302728200 12750366

[B12] Feng G. Goodridge H. S. Harnett M. M. Wei X. Nikolaev A. V. Higson A. P. (1999). Extracellular signal-related kinase (ERK) and p38 mitogen-activated protein (MAP) kinases differentially regulate the lipopolysaccharide-mediated induction of inducible nitric oxide synthase and IL-12 in macrophages: *Leishmania phosphoglycans* subvert macr. J. Immunol. 163, 6403–6412. 10.4049/jimmunol.166.3.1912 10586030

[B13] Gabriel C. McMaster W. R. Girard D. Descoteaux A. (2010). *Leishmania donovani* promastigotes evade the antimicrobial activity of neutrophil extracellular traps. J Immunol. 185, 4319–4327. 10.4049/jimmunol.1000893 20826753

[B14] Gazzinelli R. T. Ropert C. Campos M. A. (2004). Role of the Toll/interleukin-1 receptor signaling pathway in host resistance and pathogenesis during infection with protozoan parasites. Immunol Rev. 201, 9–25. 10.1111/j.0105-2896.2004.00174.x 15361229

[B15] Guimarães L. H. Machado P. R. L. Lago E. L. Morgan D. J. Schriefer A. Bacellar O.. (2009). Atypical manifestations of tegumentary leishmaniasis in a transmission area of *Leishmania braziliensis* in the state of Bahia, Brazil. Trans. R. Soc. Trop. Med. Hyg. 103, 712–715. 10.1016/j.trstmh.2009.04.019 19481233 PMC2714265

[B16] Guimarães L. H. Queiroz A. Silva J. A. Silva S. C. Magalhães V. Lago E. L.. (2016). Atypical manifestations of cutaneous leishmaniasis in a region endemic for *Leishmania braziliensis*: clinical, immunological and parasitological aspects. PLoS Negl. Trop. Dis. 10:e0005100. 10.1371/journal.pntd.0005100 27906988 PMC5131895

[B17] Guimaraes-Costa A. B. Nascimento M. T. C. Froment G. S. Soares R. P. P. Morgado F. N. Conceicao-Silva F.. (2009). *Leishmania amazonensis* promastigotes induce and are killed by neutrophil extracellular traps. Proc. Natl. Acad. Sci. U.S.A. 106, 6748–6753. 10.1073/pnas.0900226106 19346483 PMC2672475

[B18] Holm Å. V. Tejle K. Magnusson K. E. Descoteaux A. Rasmusson B. (2001). *Leishmania donovani* lipophosphoglycan causes periphagosomal actin accumulation: correlation with impaired translocation of PKCα and defective phagosoem maturation. Cell. Microbiol. 3, 439–447. 10.1046/j.1462-5822.2001.00127.x 11437830

[B19] Ibraim I. C. De Assis R. R. Pessoa N. L. Campos M. A. Melo M. N. Turco S. J.. (2013). Two biochemically distinct lipophosphoglycans from *Leishmania braziliensis* and *Leishmania infantum* trigger different innate immune responses in murine macrophages. Parasites Vectors 6, 1–11. 10.1186/1756-3305-6-54 23497381 PMC3606350

[B20] Ilg T. Etges R. Overath P. McConville M. J. Thomas-Oates J. Thomas J.. (1992). Structure of *Leishmania mexicana* lipophosphoglycan. J. Biol. Chem. 267, 6834–6840. 1551890

[B21] Lima J. B. Araújo-Santos T. Lázaro-Souza M. Carneiro A. B. Ibraim I. C. Jesus-Santos F. H.. (2017). *Leishmania infantum* lipophosphoglycan induced-Prostaglandin E2 production in association with PPAR-γ expression via activation of Toll like receptors-1 and 2. Sci. Rep. 7:14321. 10.1038/s41598-017-14229-8 29084985 PMC5662570

[B22] Luz N. F. Andrade B. B. Feijo D. F. Araujo-Santos T. Carvalho G. Q. Andrade D.. (2012). Heme oxygenase-1 promotes the persistence of *Leishmania chagasi* infection. J. Immunol. 188, 4460–4467. 10.4049/jimmunol.1103072 22461696 PMC3331931

[B23] Macedo D. H. Menezes-Neto A. Rugani J. M. Rocha A. C. Silva S. O. Melo M. N.. (2016). Low frequency of LRV1 in *Leishmania braziliensis* strains isolated from typical and atypical lesions in the State of Minas Gerais, Brazil. Mol. Biochem. Parasitol. 210, 50–54. 10.1016/j.molbiopara.2016.08.005 27546549 PMC5125831

[B24] Mahoney A. B. Sacks D. L. Saraiva E. Modi G. Turco S. J. (1999). Intra-species and stage-specific polymorphisms in lipophosphoglycan structure control *Leishmania donovani*—Sand fly interactions. Biochemistry 38, 9813–9823. 10.1021/bi990741g 10433687

[B25] Nogueira P. M. Assis R. R. Torrecilhas A. C. Saraiva E. M. Pessoa N. L. Campos M. A. (2016). Lipophosphoglycans from *Leishmania amazonensis* strains display immunomodulatory properties via TLR4 and do not affect sand fly infection. PLoS Negl. Trop. Dis. 10, 1–17. 10.1371/journal.pntd.0004848 PMC4980043 27508930

[B26] Nogueira P. M. Guimarães A. C. Assis R. R. Sadlova J. Myskova J. Pruzinova K. (2017). *Lipophosphoglycan polymorphisms* do not affect *Leishmania amazonensis* development in the permissive vectors *Lutzomyia migonei* and *Lutzomyia longipalpis*. Parasites Vectors 10:608 10.1186/s13071-017-2568-8 29246180 PMC5732482

[B27] Paranaíba L. F. De Assis R. R. Nogueira P. M. Torrecilhas A. C. Campos J. H. Silveira A. C. D. O.. (2015). *Leishmania enriettii*: biochemical characterisation of lipophosphoglycans (LPGs) and glycoinositolphospholipids (GIPLs) and infectivity to *Cavia porcellus*. Parasites Vectors 8, 1–14. 10.1186/s13071-015-0633-8 25595203 PMC4311450

[B28] Passero L. F. D. Assis R. R. da Silva T. N. F. Nogueira P. M. Macedo D. H. Pessoa N. L.. (2015). Differential modulation of macrophage response elicited by glycoinositolphospholipids and lipophosphoglycan from *Leishmania (Viannia) shawi*. Parasitol. Int. 64, 32–35. 10.1016/j.parint.2015.01.006 25619843

[B29] Quaresma P. F. Ferreira C. Marteleto J. Rugani N. Freire J. D. M. Baptista R. D. P.. (2018). Distinct genetic profiles of *Leishmania (Viannia) braziliensis* associate with clinical variations in cutaneous-leishmaniasis patients from an endemic area in Brazil. Parasitology 145, 1161–1169. 10.1017/S0031182018000276 29526166

[B30] Rugani J. N. Quaresma P. F. Gontijo C. F. Soares R. P. Monte-Neto R. L. (2018). Intraspecies susceptibility of *Leishmania (Viannia) braziliensis* to antileishmanial drugs: antimony resistance in human isolates from atypical lesions. Biomed. Pharmacother. 108, 1170–1180. 10.1016/j.biopha.2018.09.149 30372818

[B31] Sacks D. L. Pimenta P. F. McConville M. J. Schneider P. Turco S. J. (1995). Stage-specific binding of *Leishmania donovani* to the sand fly vector midgut is regulated by conformational changes in the abundant surface lipophosphoglycan. J. Exp. Med. 181, 685–697. 10.1084/jem.181.2.685 7836922 PMC2191891

[B32] Sallusto F. Zielinski C. E. Lanzavecchia A. (2012). Human Th17 subsets. Eur. J. Immunol. 42, 2215–2220. 10.1002/eji.201242741 22949319

[B33] Soares R. P. Margonari C. Secundino N. C. MacÊdo M. E. Da Costa S. M. Rangel E. F.. (2010). Differential midgut attachment of *Leishmania (Viannia) braziliensis* in the sand flies *Lutzomyia (Nyssomyia) whitmani* and *Lutzomyia (Nyssomyia) intermedia*. J. Biomed. Biotechnol. 2010:827851. 10.1155/2010/439174 20011070 PMC2789580

[B34] Soares R. P. P. Barron T. McCoy-Simandle K. Svobodova M. Warburg A. Turco S. J. (2004). *Leishmania tropica*: intraspecific polymorphisms in lipophosphoglycan correlate with transmission by different Phlebotomus species. Exp. Parasitol. 107, 105–114. 10.1016/j.exppara.2004.05.001 15208044

[B35] Soares R. P. P. Cardoso T. L. Barron T. Araújo M. S. S. Pimenta P. F. P. Turco S. J. (2005). *Leishmania braziliensis*: a novel mechanism in the lipophosphoglycan regulation during metacyclogenesis. Int. J. Parasitol. 35, 245–253. 10.1016/j.ijpara.2004.12.008 15722076

[B36] Soares R. P. P. Macedo M. E. Ropert C. Gontijo N. F. Almeida I. C. Gazzinelli R. T.. (2002). *Leishmania chagasi*: lipophosphoglycan characterization and binding to the midgut of the sand fly vector Lutzomyia longipalpis. Mol. Biochem. Parasitol. 121, 213–224. 10.1016/S0166-6851(02)00033-6 12034455

[B37] Spath G. F. Garraway L. A. Turco S. J. Beverley S. M. (2003). The role(s) of lipophosphoglycan (LPG) in the establishment of *Leishmania major* infections in mammalian hosts. Proc. Natl. Acad. Sci. U.S.A. 100, 9536–9541. 10.1073/pnas.1530604100 12869694 PMC170953

[B38] Tavares N. M. Araújo-Santos T. Afonso L. Nogueira P. M. Lopes U. G. Soares R. P.. (2014). Understanding the mechanisms controlling *Leishmania amazonensis* infection *in vitro*: the role of LTB4 derived from human neutrophils. J. Infect. Dis. 210, 656–666. 10.1093/infdis/jiu158 24634497 PMC4111911

[B39] Tolson D. L. Turco S. J. Beecroft R. P. Pearson T. W. (1989). The immunochemical structure and surface arrangement of *Leishmania donovani* lipophosphoglycan determined using monoclonal antibodies. Mol. Biochem. Parasitol. 35, 109–118. 10.1016/0166-6851(89)90113-8 2475775

[B40] Tuon F. F. Amato V. S. Bacha H. A. Almusawi T. Duarte M. I. Amato Neto V. (2008). Toll-like receptors and leishmaniasis. Infect. Immun. 76, 866–872. 10.1128/IAI.01090-07 18070909 PMC2258802

[B41] Turco S. J. Descoteaux A. (1992). The lipophosphoglycan of Leishmania parasites. Annu. Rev. Microbiol. 46, 65–94. 1444269 10.1146/annurev.mi.46.100192.000433

[B42] Vinet A. F. Fukuda M. Turco S. J. Descoteaux A. (2009). The *Leishmania donovani* lipophosphoglycan excludes the vesicular proton-ATPase from phagosomes by impairing the recruitment of Synaptotagmin V. PLoS Pathog. 5:e1000628. 10.1371/journal.ppat.1000628 19834555 PMC2757729

